# Prevalence of intestinal parasites, salmonella and shigella among apparently health food handlers of Addis Ababa University student’s cafeteria, Addis Ababa, Ethiopia

**DOI:** 10.1186/s13104-014-0967-x

**Published:** 2015-01-24

**Authors:** Addis Aklilu, Daniel Kahase, Mekonnen Dessalegn, Negatu Tarekegn, Saba Gebremichael, Seyfe Zenebe, Kassu Desta, Gebru Mulugeta, Yeshiwodim Mamuye, Mohammedaman Mama

**Affiliations:** Medicine and Health Sciences College, Arba Minch University, Arba Minch, Ethiopia; Health Sciences College, Wolkite University, Wolkite, Ethiopia; Amsalu Higher Clinic, Addis Ababa, Ethoiopia; Pawi General Hospital, Addis Ababa, Ethiopia; International Clinical Laboratories, Addis Ababa, Ethiopia; Ras Desta General Hospital, Addis Ababa, Ethiopia; College of Allied Health Sciences, Addis Ababa University, Addis Ababa, Ethiopia; St. Paul’s Hospital Mellinium Medical College, Addis Ababa, Ethiopia

**Keywords:** Intestinal parasites, Salmonella, Shigella, Food handlers

## Abstract

**Background:**

Food contamination may occur at any point during its journey through production, processing, distribution, and preparation. The risk of food getting contaminated depends largely on the health status of the food handlers, their personal hygiene, knowledge and practice of food hygiene. Food borne diseases are a public health problem in developed and developing countries like Ethiopia.

**Method:**

A cross sectional study was conducted among food handlers in Addis Ababa student’s cafeteria from January to May 2013. Structured questionnaire was used to collect socio demographic data and associated risk factors. Stool specimens were examined for bacteria and intestinal parasites following standard procedures. Biochemical tests were done to identify the species of bacterial isolates. Sensitivity testing was done using Kirby- Baur disk diffusion method.

**Result:**

A total of 172 food handlers were enrolled in the study. The majority of study participants were females 134 (77.9%). About 78 (45.3%) of food handlers were found to be positive for different intestinal parasites with the most abundant parasite of Entameoba histolytica/dispar 68 (70.8%) followed by Giardia lamblia 18 (18.8%), Taenia species 5 (5.2%), Ascaris lumbricoides 2 (2.1%), hookworm 2 (2.1%) and Trichuris trichiura 1 (1.1%). Stool cultures revealed 3.5% of Salmonella isolates (Sero-grouping on Salmonella isolate was not done), while Shigella species was not isolated from any of the stool samples obtained from Food handlers. All isolates of Salmonella were sensitive to ciprofloxacin, amikacin and gentamicin but resistant to ampicillin, clindamycin, and erythromycin.

**Conclusion:**

The present study revealed a high prevalence of intestinal parasite in asymptomatic (apparently health) food handlers. Such infected food handlers can contaminate food, drinks and could serve as source of infection to consumers via food chain.

## Background

Food contamination may occur at any point during its journey through production, processing, distribution, and preparation. The risk of food getting contaminated depends largely on the health status of the food handlers, their personal hygiene, knowledge and practice of food hygiene [1].

There are more than 250 different food borne diseases in world wide. Most of these diseases are infectious, caused by a variety of bacteria, viruses, and parasites. Other food borne diseases can be poisonings, caused by harmful toxins or chemicals like poisonous mushrooms and enterotoxins of some bacteria. Candidate bacteria for this are *Salmonella*, *Campylobacter*, *Listeria*, pathogenic *Escherichia coli (E. coli)*, *Yersinia*, *Shigella*, *Enterobacter* and *Citrobacter* [2]. These organisms may exist on food handler’s skin, from which it may be transmitted to cooked moist protein-rich foods, and become intoxication agents if these foods are then kept for several hours without refrigeration or stored in containers [3].

Intestinal parasites and enteropathogenic bacteria are transmitted directly and indirectly through objects such as food, water, nails, fingers, etc. Compared to other parts of the hand, fingernails harbors the most microorganisms and difficult to clean easily [4].

The World health organization (WHO) estimated that in developed countries, up to 30% of the population suffers from food borne diseases each year, whereas in developing countries up to 2 million deaths are estimated per year [5].

It is estimated that 3.5 billion people are affected and that 450 million people are ill as a result of intestinal parasites and protozoan infections, majority of being children [4].

Diarrheal diseases, mostly caused by food borne or water borne microbial pathogens, are leading causes of illness and deaths in developing countries, killing an estimated 1.9 million people annually at the global level. Even in developed countries, an estimated one-third of the populations are affected by microbiological food borne diseases each year [6].

According to Centers for Disease Control (CDC) food-borne diseases cause an estimated 76 million illnesses, 325,000 hospitalizations, and 5,000 deaths in the U.S. each year. The cost of the most common food borne illnesses in the United States is estimated at $6.5–$34.9 billion annually [2].

Therefore, the results of this study were contributed in provision of concrete evidence about the *Salmonella*, *Shigella*, and intestinal parasites prevalence rate among food handlers and antibiotic sensitivity profile of the isolated bacteria in the study area.

## Methods

### Study area and period

The study was carried out on asymptomatic food handlers (those participated in food preparation, dispatch and store) of Addis Ababa University student’s cafeteria from January to May 2013. A total of 1000 food handlers are working in this student’s cafeteria. Addis Ababa University (AAU) is located in Addis Ababa, the capital city of Ethiopia.

### Study design

A Cross sectional study design was conducted among asymptomatic food handlers.

### Study population

All individual working as a food handler in student’s cafeteria of Addis Ababa University.

### Eligibility criteria

#### Inclusion criteria

Food handlers working in Addis Ababa University student’s cafeterias and given informed consent were included in the study.

### Exclusion criteria

Food handlers who have diarrhea, fever, taking antibiotics, antihelminthics and incomplete questionnaires were excluded from the study.

### Sample size determination and sampling technique

#### Sample size determination

The sample size was determined by using a single population proportion formula considering the following assumptions: Zα/2 = 1.96 for the standard scale of 95% level of confidence, level of precision = 5%, P = 0.21:

$$ n=\frac{{\left({z}_{\propto /2}\right)}^2*P\left(1-P\right)}{d^2} = 253 $$

Since the total number of the source population was 1000 and below 10,000 a correction formula was used to adjust the sample size as follows:

$$ \frac{n}{1+\frac{n}{N}}=\frac{253}{1+\frac{253}{1000}}\approx 200 $$

### Sampling technique

Simple random sampling technique using lottery method was used to select the study subjects. Complete list of food handlers was obtained from human resource management of Addis Ababa University.

### Data collection and laboratory processing

Socio-demographic data was gathered by using structured pretested questionnaire and face to face interview. Stool specimens were collected from food handlers with a suitable labeled wide-mouthed plastic container and clean wooden applicator stick. Specimens were immediately transported to laboratory within 2–4 hours using ice box.

### Direct smear examination for stool samples

On a microscope slide, about 1–2 mg of stool was emulsified in a drop of normal saline (0.85% NaCl) on the left hand side of the slide, and in Lugol’s iodine on the right side of the slide. A cover-slip was then placed on each side, and the slides were scanned under 10× and 40× objective lenses of a light microscope, as required. Saline direct smear is used mainly for detection of motility of intestinal protozoan trophozoites, which are seen in liquid or semi-liquid specimens. Iodine direct smear shows the characteristic features of the diagnostic stages in more details and formol ether sedimentation concentration technique was used for detection of cysts, ova and larvae.

### Culture and identification

All stool specimens was inoculated into Selenite F broth (Oxoid, UK) and incubated for 24 hours at 37°C followed by subculture on Xylose Lysine Deoxycholate (XLD) (Oxoid) at 37°C for 24 hours for isolation of *Shigella* and *Salmonella* species. Biochemical tests were performed on colonies from primary cultures for final identification of the isolates. The bacteria were identified by performing a series of biochemical tests (Oxoid). Namely, Kliger iron agar, indole, Simon’s citrate agar, lysine iron agar, urea, oxidase and motility. The test organism was uniformly seeded over the Mueller-Hinton agar (oxoid) surface and exposed to a concentration gradient of antibiotic diffusing from antibiotic-impregnated paper disk into the agar medium, and then incubated at 37°C for 16–18 hours. Organisms’ sensitive to the antibiotic were inhibited from growing in a circular zone around the antibiotic impregnated paper disk. Diameters of the zone of inhibition around the discs were measured to the nearest millimeter using a ruler and classified as sensitive, intermediate, and resistant according to the standardized table supplied by CLSI, 2011. All intermediate readings were taken as sensitive during data entry. The drugs tested were ciprofloxacin (5 μg), Trimethoprim-sulfamethoxazole (25 μg), ampicillin (10 μg), gentamicin (10 μg), amikacin (30 μg), doxycycline (30 μg), cefotaxime (5 μg), clindamycin (2 μg), amoxicillin (30 μg) and erythromycin (15 μg).

### Statistical analysis

Data was edited, cleaned, entered and analyzed using statistical package for social science (SPSS) version 20. Descriptive analysis such as frequencies and mean were used. Initially the association between each exposure and the presence of infection was assessed using the chi-square test, and odds ratio was computed to measure the strength of the association. P-value of ≤ 0.05 was considered to indicate statistically significant differences. The result was presented using tables and charts.

### Ethical consideration

Ethical clearance was obtained from Ethical Review Committee, Faculty of Medicine; Addis Ababa University. Written informed consent was obtained from study participants. Strict confidentiality was maintained during the interview process as well as anonymity was kept during data processing and report writing. Food handlers who have found to be positive for enteric pathogens were referred to their respective staff medical center for appropriate antimicrobial treatments.

## Results

### Sociodemographic data

A total of 172 asymptomatic food handlers were included in the study with a response rate of 86%. One hundred thirty four (77.9%) of the participants were females. The mean age was 35 years with a standard deviation of 11 and ranging from 17–75 years. The majority of food handlers 97 (56.4%) were in the age group of 17–34. Majority of the study participants were completed secondary school 84 (48.8%) (Table 1).

**Table 1:** **Socio demographic characteristics of food-handlers in AAU student’s cafeteria, Addis Ababa, January to May 2013**

**Socio demographic data**	**Frequency**	**Percentage**
**Age (years)**		
17–34	97	56.4
35–52	55	32.0
>53	20	11.6
**Sex**		
Male	38	22.1
Female	134	77.9
**Educational status**		
Illiterate	5	2.9
Primary	59	34.3
Secondary	84	48.8
Higher	24	14

### Prevalence of intestinal parasites, Salmonella and Shigella species

#### Prevalence of intestinal parasites

**Figure 1:**
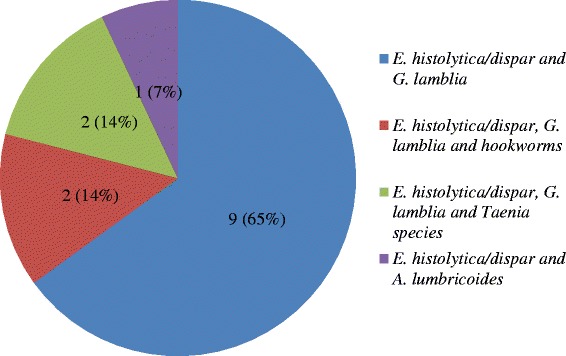
**Prevalence of mixed parasitic infections in food handlers in AAU student’s cafeteria, Addis Ababa, January to May 2013.**

Of 172 stool specimens, 78 (45.3%) were found to be positive for different intestinal parasites. The most abundant parasite was *Entameoba histolytica/dispar* (*E. histolytica/dispar*) 68 (70.8%) followed by *Giardia lamblia* (*G. lamblia*) 18 (18.8%), *Taenia* species 5 (5.2%), *Ascaris lumbricoides* (*A. lumbricoides*) 2 (2.1%), *Trichuris trichiura* (*T. trichiura*) 1 (1.1%) and *hookworm* 2 (2.1%) (Table 2). Among 78 positive food handler’s, 18 (18.8%) had mixed infections. The dominant parasites among mixed infections were *E. histolytica* and *G. lamblia* (Figure 1).

**Table 2:** **Type and prevalence of intestinal parasites isolated from stool specimens of food handlers in AAU student’s cafeteria, Addis Ababa, January to May 2013**

**Type of parasites**	**Frequency**	**Percentage**
*E. histolytica/dispar*	68	70.8
*G. lamblia*	18	18.8
*Taenia* species	5	5.2
*A. lumbricoides*	2	2.1
Hookworms	2	2.1
*T. trichiura*	1	1.1
Total	96	100

### Prevalence of *Shigella* and *Salmonella* species

Out of 172 food-handlers screened, stool cultures revealed only six (3.5%) *Salmonella* isolates. These bacterial isolates were identified from food handlers who did not have regular medical checkup. No *Shigella* species was isolated from any of the stool samples obtained from Food handlers.

### Associated risk factors

Of the total food handlers 122 were worked for greater than 2 years, of those that worked longer than 2 years, 61 had parasites and three of them are positive for *Salmonella* species. Even though, one hundred thirty nine food handlers (139) wash their hands only with water after toilet, 65 of them harbor parasite while five of them were positive for *Salmonella* species. From food handlers who did not take food preparation training, sixty six (66) had different intestinal parasites. The number of parasites found in food handlers who experience regular medical checkup does not have statistical association with that of who do not. In general Parasite and *Salmonella* asymptomatic carriers did not have any significant association with those checked risk factors.

### Antimicrobial susceptibility pattern of *Salmonella* isolates

The susceptibility of Salmonella was tested against 10 selected antimicrobial agents as followed in Tables 3 and 4. The results obtained showed that the organisms varied in their susceptibility to all the antimicrobials used. All of them showed multi-resistances (resistance to two or more than two classes of antimicrobials) to the antibiotics. Percentage of isolates that were resistant to ampicillin, clindamycin, amoxicillin and erythromycin were 100%, followed by sulphamethoxazole trimethoprim and cefotaxime which were 16.7%. All isolates were 100% susceptible to Ciprofloxacin, gentamicin and amikacin.

**Table 3:** **Anti-microbial susceptibility patterns of** ***Salmonellae*** **Species from food handlers in AAU student’s cafeteria, Addis Ababa, and January to May 2013**

**Antibiotics**	***Salmonella***			
	**R**		**S**	
	**NO**	**%**	**NO**	**%**
Ciprofloxacin (5 μg)	0	0	6	100
Trimethoprim (25 μg)	1	16.7	5	83.3
Ampicillin (10 μg)	6	100	0	0
Gentamicin (10 μg)	0	0	6	100
Amikacin (30 μg)	0	0	6	100
Doxycycline (30 μg)	0	0	6	100
Cefotaxime (5 μg)	1	16.7	5	83.3
Clindamycin (2 μg)	6	100	0	0
Amoxicillin (30 μg)	6	100	0	0
Erythromycin (15 μg)	6	100	0	0

**Table 4:** **Anti biogram pattern of isolated** ***Salmonellae*** **species from food handlers in AAU student’s cafeteria, Addis Ababa, and January to May 2013**

**Drug**	**Bacteria isolates**	**Count**
AM^R^ AMP^R^ E^R^ SXT^R^ DA^R^	*Salmonella*	1
AM^R^ AMP^R^ E^R^ DA^R^	*Salmonella*	1
AM^R^ AMP^R^ E^R^ DA^R^ CXT^R^	*Salmonella*	1
AM^R^ AMP^R^ E^R^ DA^R^	*Salmonella*	3

Key: R: −resistance, AM: -amoxicillin, AMP: − ampicillin, E: − erythromycin, SXT:- Sulphamethoxazole-trimethoprim, CXT: - cefotaxime, DA: - Clindamycin.

## Discussion

Food handlers may be carrying a wide range of enteropathogens and have been implicated in the transmission of many infections to the public in the community and to patients in hospitals. The spread of disease via food handlers is a common and persistent problem worldwide. Therefore, this study was undertaken to assess prevalence of intestinal parasites, *Salmonella* and *Shigella* among food handlers of Addis Ababa University Student’s Cafeteria, Addis Ababa, Ethiopia.

In this study 45.3% and 3.5% of the food handlers were positive for intestinal parasites and *Salmonella*, respectively. The high prevalence of intestinal parasites (45.3%) in this study among food-handlers was in agreement with the findings of other studies conducted in Ethiopia like Bahir Dar Town (41.1%) [6] and elsewhere in Venezuela, Zulia state (48.7%) [7], Minas Gerais, Brazil (47.1%) [8] and Irbid, Jordan (48.0%) [9]. The higher rates in this study may be attributed to improper hygiene of food handlers.

Higher prevalence of intestinal parasites were reported in Ethiopia from Hawassa, (63%) [10], Mekele University 49.4% [11], Uberlandia and Sanliurfa, Southeastern Anatolia (52.2%) [12], Abeokuta, Nigeria (97%) [13] when compared with present study, however lower prevalence was reported in North west Ethiopia (29.1%) [5] and Khuzestan, Southwest of Iran (7.78%) [14], North India (1.3 to 7%) [15], Turkey (8.8%) [16], Thailand 10.3% [17], Makkah 31.94% [18], Gaza Strip, Palestine (24.3%) [19], Khartoum, Sudan (29.4%) [20], Omdurman, Sudan (30.1%) [3]. This discrepancy may be largely due to epidemiological, environmental distribution difference, poor personal hygiene practices, environmental sanitation and ignorance of health-promotion practices.

In this study the dominant parasite from food handlers were *E. histolytica/dispar* 68 (70.8%) followed by *G. lamblia* 18 (18.8%). This was similar with the work of Selman CA [16] Turkey who identified *E. histolytica* (69.9%) and *G. lamblia* (24.6%) as commonest parasite, while study done in Ethiopia also identified (*E. histolytica/dispar*) 12.8% and *G. lamblia* 7. 0% as the most leading parasite [6], but study conducted in Hawassa town [10], Ethiopia, Zulia State (13.4%) [7], Kahuzestan, Southwest Iran (4.5%) [14] identified *G. lamblia* as the leading parasite followed by other parasites.

In the present study six *Salmonella* (3.5%) was isolated from stool culture of food handlers, similarly study done in Kumasi, Ghana among food vendors of 258 identified typhoidal *Salmonellae* from six people, giving a carriage rate of 2.3% [21]. This slight difference may be due to large number of participants involved in Kumasi, Ghana study when compared with present study.

Study conducted in Tamilnadu, India identified 65.7% and 79% *Salmonella* from finger nail contents and nail cuts respectively among hotel works which is alarming and indicated that main sources of pathogen transfer such as *Salmonella* is through improper hand washing [22].

There are also studies in agreement with present study in identifying low prevalence of *Salmonella* in food handlers like study done in Ethiopia, Bahir Dar Town (1.6%) [6] and elsewhere in Irbid, Jordan (6%) [9], Omdurman, Sudan (1.3%) [3].

In the present study, no *Shigella* species were recovered from stool culture of food handlers. However, *Shigella* was the most common among enteropathogens isolated among food handlers in a tertiary care hospital of North India (13.3%) [15].

This study indicated that no statistically significant association between the frequency of parasitic and bacterial infection and age, sex, service year, hand washing habit after toilet, food preparation training, finger nail status and medical checkup. This may be due to low sample size. Similarly, finding in Khartoum, Sudan showed that no statistical significant association particularly with parasitic infection comparable for age, sex and service year [20].

All isolates were resistant at least to three of antimicrobials tested and one isolate was resistant to trimethoprim. Study done in Bahir Dar showed Salmonella typhi (S. Typhi) had high resistances against ampicillin, cotrimoxazole, tetracycline, chloramphenicol, gentamicin and norfloxacillin indicated that antimicrobial resistance of *S. typhi* is an increasing concern [6].

## Conclusion

Majority of the food-handlers (56.4%) were young adults aged 17–34 years. Forty five percent (45.3%) stool specimens were positive for different intestinal parasites. *Salmonella* isolation rate was 3.5% of which 100% resistance to ampicillin, amoxicillin, erythromycin and 100% sensitive to ciprofloxacin, gentamicin and amikacin. The present study revealed a high prevalence of intestinal parasite in asymptomatic (apparently health) food handlers. Such infected food handlers can contaminate food, drinks and could serve as source of infection to consumers via food chain.
